# Predicting worsening heart failure hospitalizations in patients with implantable cardioverter defibrillators: is it all about alerts? A pooled analysis of nine trials

**DOI:** 10.1093/europace/euae032

**Published:** 2024-01-31

**Authors:** Giovanni Luca Botto, Gianfranco Sinagra, Alan Bulava, Alessio Gargaro, Tobias Timmel, Daniele Giacopelli, Antonio D’Onofrio, Laurence Guédon-Moreau

**Affiliations:** U.O. Electrophysiology, ASST Rhodense, 95 Viale Carlo Forlanini, 20024 Garbagnate Milanese (MI), Italy; Cardiothoracovascular Department, Cattinara Hospital, ASUGI and University of Trieste, Trieste, Italy; Faculty of Health and Social Sciences, Ceske Budejovice Hospital, University of South Bohemia in Ceske Budejovice, Ceske Budejovice, Czech Republic; Clinical Unit, Biotronik Italia S.P.A., Cologno Monzese (MI), Italy; Center for Clinical Research, Biotronik SE & Co. KG, Berlin, Germany; Clinical Unit, Biotronik Italia S.P.A., Cologno Monzese (MI), Italy; Unità Operativa di Elettrofisiologia, Studio e Terapia delle Aritmie, Monaldi Hospital, Naples, Italy; CHU Lille, University of Lille, Lille University Hospital Center, Lille, Hauts-de-France, France

**Keywords:** Worsening heart failure hospitalization, Predictive algorithm, Heart failure score, Implantable defibrillators, Remote monitoring, Heart rate

## Abstract

**Aims:**

To predict worsening heart failure hospitalizations (WHFHs) in patients with implantable defibrillators and remote monitoring, the HeartInsight algorithm (Biotronik, Berlin, Germany) calculates a heart failure (HF) score combining seven physiologic parameters: 24 h heart rate (HR), nocturnal HR, HR variability, atrial tachyarrhythmia, ventricular extrasystoles, patient activity, and thoracic impedance. We compared temporal trends of the HF score and its components 12 weeks before a WHFH with 12-week trends in patients without WHFH, to assess whether trends indicate deteriorating HF regardless of alert status.

**Methods and results:**

Data from nine clinical trials were pooled, including 2050 patients with a defibrillator capable of atrial sensing, ejection fraction ≤ 35%, NYHA class II/III, no long-standing atrial fibrillation, and 369 WHFH from 259 patients. The mean HF score was higher in the WHFH group than in the no WHFH group (42.3 ± 26.1 vs. 30.7 ± 20.6, *P* < 0.001) already at the beginning of 12 weeks. The mean HF score further increased to 51.6 ± 26.8 until WHFH (+22% vs. no WHFH group, *P* = 0.003). As compared to the no WHFH group, the algorithm components either were already higher 12 weeks before WHFH (24 h HR, HR variability, thoracic impedance) or significantly increased until WHFH (nocturnal HR, atrial tachyarrhythmia, ventricular extrasystoles, patient activity).

**Conclusion:**

The HF score was significantly higher at, and further increased during 12 weeks before WHFH, as compared to the no WHFH group, with seven components showing different behaviour and contribution. Temporal trends of HF score may serve as a quantitative estimate of HF condition and evolution prior to WHFH.

## Graphical Abstract

**Graphical Abstract:**
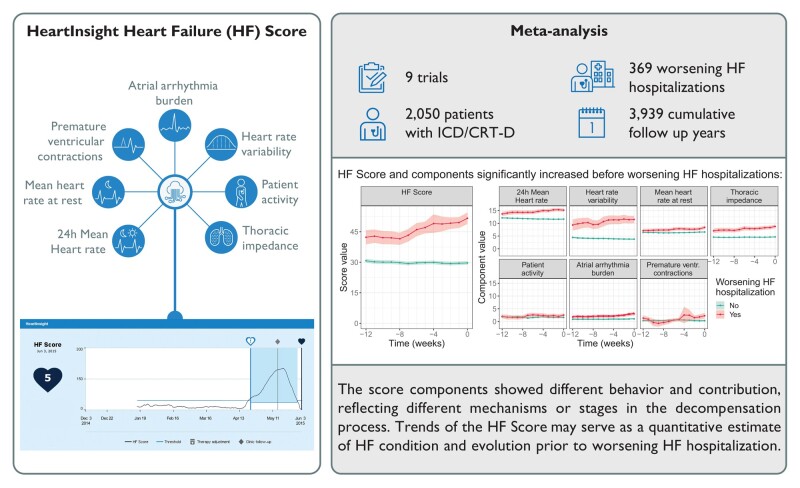

**What’s new?**

- Data of 2050 ICD or CRT-D patients from nine clinical trials were pooled to compare 12-week trends of a remote monitoring-based multiparametric heart failure (HF) score before 369 worsening HF hospitalizations (WHFH), with trends in patients without WHFH.
- The mean HF score was significantly higher at 12 weeks before WHFH than in the no WHFH group, and it further increased by 22% until a WHFH event.
- The seven algorithm components showed different behaviour and contribution, reflecting different mechanisms or different stages in the decompensation process: as compared to the no WHFH group, 24 h heart rate (HR), HR variability, and thoracic impedance were already higher 12 weeks before WHFH, with 71% contribution to the HF score; nocturnal HR, atrial tachyarrhythmia, ventricular extrasystoles, and patient activity significantly increased until WHFH with total 31% contribution to the HF score.
- The HF score may serve as quantitative estimate of HF condition prior to WHFH.

## Introduction

Routine ambulatory care is insufficient to prevent acute heart failure leading to functional disability, reduced quality of life, increased mortality, and high socioeconomic costs, particularly those associated with hospitalizations for worsening heart failure (WHF).^1–4^ In the recent Selection of Potential Predictors of Worsening Heart Failure (SELENE HF) study, an algorithm for prediction of WHF hospitalization was developed and validated in patients with implantable cardioverter-defibrillators (ICDs) and cardiac resynchronization therapy defibrillators (CRT-Ds).^5^ The algorithm combined temporal trends of physiologic parameters obtained by automatic daily remote monitoring (e.g. heart rate, arrhythmia burden, physical activity, and thoracic impedance) with an optional baseline risk-stratiﬁer (Seattle HF Model) into a single heart failure (HF) index.^5,6^ When the HF index stably exceeds a pre-specified threshold, the algorithm generates an alert for impending hospitalization for WHF. With default settings, the sensitivity in predicting the ﬁrst post-implant hospitalization for WHF was 65.5% and the false alert rate was 0.69 per patient-year.^5^ However, deteriorating HF conditions do not necessarily lead to a hospital admission, and not all hospitalizations for WHF can be foreseen by predictive algorithms.^5,7,8^

It is therefore important to evaluate the clinical relevance of the HF index temporal trends provided by the algorithm, regardless of the alert status. Increasing index values may indicate deteriorating HF status and incorporate clinical information of interest for interpretation, medical decision-making, and timing. To examine this issue, we pooled data from nine clinical trials and analysed 12-week trends of the HF index and its components before WHF hospitalizations compared to 12-week trends in patients who did not have hospitalizations for WHF.

## Methods

### Selection of clinical trials and patients

Completed or ongoing clinical trials (database lockout 29 November 2022) were included in the present analysis if WHF events were adjudicated by external boards and the Biotronik Home Monitoring (HM) feature was enabled. The predictive algorithm was not available to the investigators during the execution of each trial. Patients contributed to the present analysis if they had a CRT-D or an ICD capable of atrial sensing (a dual-chamber ICD or a DX ICD with a floating atrial dipole on the ICD lead^9^), a left ventricular ejection fraction (LVEF) ≤ 35%, New York Heart Association (NYHA) class II or III HF, and no long-standing or permanent atrial fibrillation (AF) before device implantation. These requirements correspond to the SELENE HF patient inclusion criteria.^5^

### Predictive algorithm

**Figure 1:**
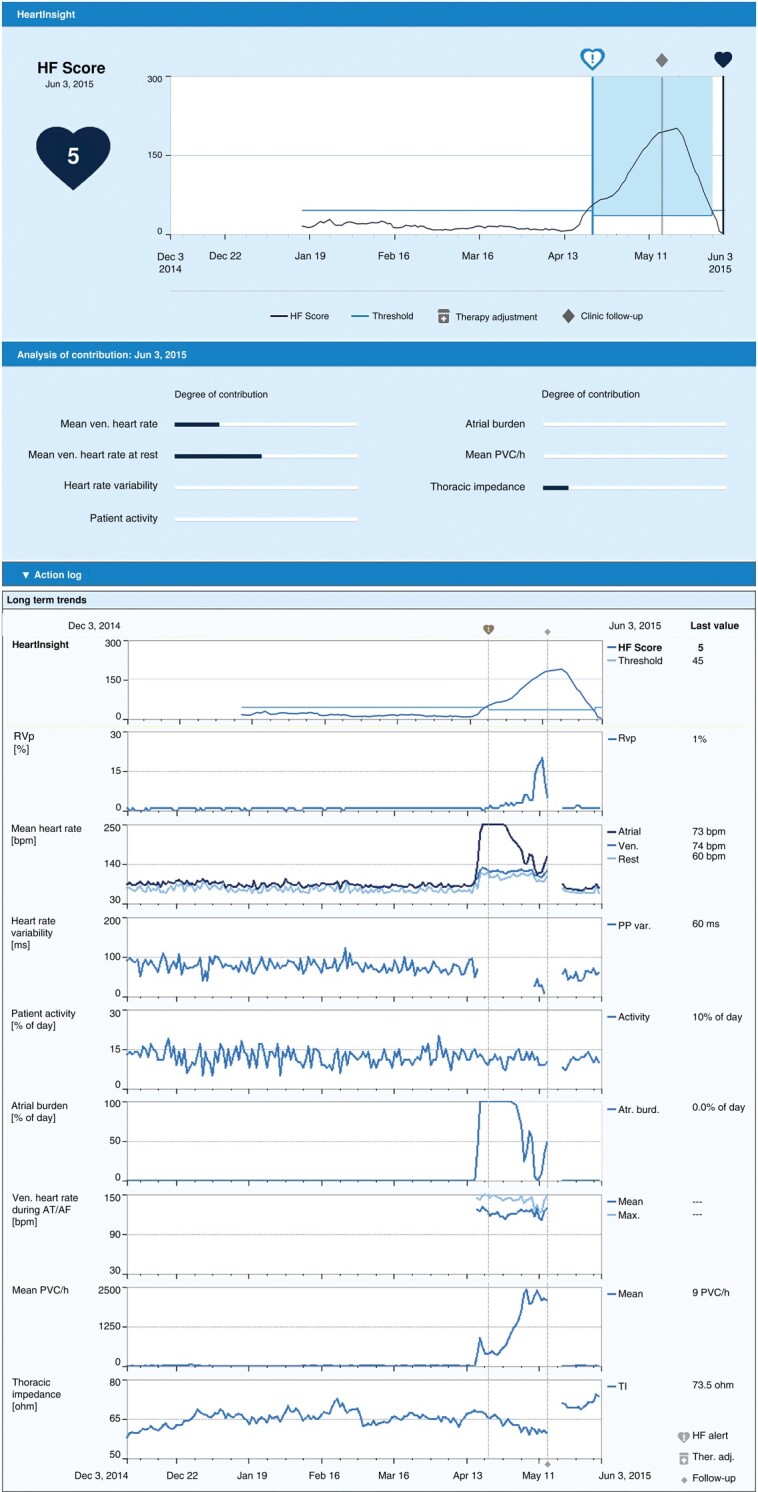
A reconstruction of the HeartInsight dashboard on the Biotronik Home Monitoring platform generated with data from a case included in our analysis. The dashboard consists of four segments. The first segment shows the current HF Score (5) calculated by the HeartInsight algorithm, temporal trend of HF score, programmed nominal threshold for alerts (default 45), recovery threshold (calculated as nominal threshold minus 10), and the date of the alert to worsening HF (April 22). The second segment visualizes contributions of dynamic algorithm components to the current HF Score. An Action log is available to keep track of the actions taken by the clinic in response to the alert. The last segment visualizes temporal trends of all available HF diagnostics, including percentage of CRT delivery (not included in HF score), mean heart rates, heart rate variability, patient activity, atrial arrhythmia burden, ventricular heart rate during atrial arrhythmia (not included in HF score), the number of PVC per hour, and thoracic impedance. AT, atrial tachycardia; AF, atrial fibrillation; BiV, biventricular; bpm, beats/minute; CRT, cardiac resynchronization therapy; HF, heart failure; HM, Home Monitoring; PVC, premature ventricular contractions; ven, ventricular.

The predictive algorithm developed as part of the SELENE HF trial has been recently integrated into the HM platform as the HeartInsight feature (Biotronik SE & Co. KG, Berlin, Germany). It calculates the HF Score daily by combining temporal trends of seven longitudinal HM parameters collected during 12 previous weeks. The parameters include monotone increase of 24 hour mean heart rate (24 h HR), instability of nocturnal mean heart rate (Night HR), monotone decrease of heart rate variability (HRV) assessed in periods without atrial high-rate episodes, burden of atrial high-rate episodes expressed in percent of 24 h (AHRE %), trend of premature ventricular contractions per hour (PVC/h), trend of patient’s physical activity (Activity), and monotone decrease of thoracic impedance (TI). When the HF Score exceeds a programmable threshold (default setting: 45), the system alerts clinical staff to an increased risk of WHF hospitalization by a standard HM notification and provides all relevant HF diagnostics, as shown in *Figure 1*. For convenience, the HF Score in HeartInsight is equal to the HF index from SELENE HF multiplied by a factor of 10.

The ‘in-alert’ status is terminated when the HF Score falls below a recovery threshold that is lower than the nominal threshold. The Seattle HF Model^6^ score obtained at device implantation is a static component used to optimize algorithm specificity. Since this score was not reported in all contributing trials, it is not included in the present analysis, which addresses the time-dependent algorithm components.

### Worsening heart failure hospitalizations

Worsening heart failure hospitalizations were defined as non-elective hospital admissions with at least one overnight stay, triggered by symptoms, signs, or objective evidence of WHF (LVEF, electrocardiogram, other instrumental evidence) and requiring administration of intravenous therapy for HF (diuretics, vasodilators, or inotropic agents). All events in the nine trials were adjudicated according to the same criteria.

### Study endpoints

Worsening heart failure hospitalizations occurring ≥30 days post-implantation (i.e. after the algorithm stabilization run-in period) and associated with a HM transmission rate ≥ 55% (the proportion of days with data transmission within previous 90 days) were endpoints contributing to this analysis. The 55% cut-off for the HM transmission rate has been integrated in the HeartInsight algorithm based on the algorithm-developing SELENE HF study.^5^ This low cut-off value in comparison to a 90% median HM transmission rate in clinical practice still ensures sufficient input information for the algorithm even with moderate HM compliance.

### Study objectives and methods

The primary objective was to analyse 12-week trends of the HF Score and its individual components before WHF hospitalizations, in comparison with respective trends prior to the last HM transmission in patients not hospitalized for WHF. Each week is represented by the average of the HF Score and its components, with Week 0 pertaining to Days 0–6, Week −1 to Days 7–13, and Week −12 to Days 84–90 before WHF hospitalization or last HM transmission.

Comparisons were performed in the entire patient cohort and in the pre-specified patient subgroups according to major baseline characteristics: age (</≥75 years), sex (female/male), body mass index (</≥30 kg/m²), history of AF (yes/no), renal insufficiency (yes/no), and ischaemic HF aetiology (yes/no), to see if there are any differences in temporal trends between the subgroups.

In case of repeat hospitalizations within <90 days, the analysis interval for the second event was the time between the two events. If the first WHF hospitalization occurred earlier than 90 days after the post-implant run-in period, the analysis interval was the available time between the run-in period and the event. Patients without WHF hospitalizations were excluded if they were followed for <90 days after the run-in period or if the HM transmission rate was <55% during 90 days prior to the last HM transmission.

All calculations in the present study were *post hoc*, based on the prospectively collected HM and WHF hospitalization data before the availability of the HeartInsight feature.

### Statistical methods

Continuous data are reported as mean ± standard deviation or median with interquartile range (IQR) if not normally distributed according to the Shapiro–Wilk’s test. Categorical data are reported as absolute and relative frequencies. Patient characteristics between subgroups were compared using Mann–Whitney *U* test (continuous data) and Pearson’s χ^2^ test (categorical data).

Temporal trends of the average values for the HF Score and its components from Week −12 to Week 0 were compared between patients with and without WHF hospitalizations using two-level nested linear random-intercept models: week, grouping variable, and week-group interaction term were the fixed effects; first-level random effects were modelled by random intercepts at patient level in order to account for inter-individual variability; the second nested level of random effects was modelled by random intercepts at event level, to account for multiple WHF hospitalizations in individual patients. The linear temporal trends were tested separately over the analysis interval and by baseline subgroups. Results were reported as *P*-values for the comparison of average values at Week −12 (model intercepts) and for the comparison of temporal linear trends from Week −12 to Week 0 (model slopes).

In all cases, a *P*-value of <0.05 was considered statistically significant. The analyses were performed using the STATA/MP 17.0 (StataCorp, TX, USA) and R (version 4.3; R Core Team 2023, https://www.R-project.org/) statistical software.

## Results

### Included clinical trials and patients

Eight completed trials (CASTLE-AF, DetectICI, EchoCRT, ECOST-CRT, effecT, HomeCARE II, J-HomeCARE II, SELENE HF) and one ongoing trial (BIO|Stream.HF) met the study inclusion criteria. Supplementary material online, *Table S1* in the Supplementary material explains acronyms, objectives, size, and duration of the studies.

The pooled patient cohort based on case report forms used in these studies included 5987 patients. According to a stepwise filtering process, 1654 patients were thereafter excluded due to unsuitable device or not activated HM function, 391 were excluded due to long-standing persistent or permanent AF, 232 for LVEF > 35%, 221 for NYHA class other than II/III, 1125 due to insufficient HM data (e.g. TI measurement programmed off), and 314 due to a <90-day follow-up after the run-in period without a WHF hospitalization. The remaining 2050 patients contributed to the analysis. Patient distribution per trial is shown in *Table 1*.

**Table 1:** Number of patients and WHF hospitalizations per clinical trial

Clinical trial	Time period Months/years	Patients^a^ *n* (% of total)	Patients with WHF hospitalizations^b^ *n* (% of total)	Number of WHF hospitalizations^b^ *n* (% of total)
SELENE HF	05/2012–02/2017	691 (33.7)	80 (30.9)	112 (30.4)
BIO|Stream.HF	05/2018–ongoing	408 (19.9)	24 (9.3)	30 (8.1)
EchoCRT	08/2008–03/2013	269 (13.1)	38 (14.7)	64 (17.3)
ECOST-CRT	02/2017–10/2020	205 (10.0)	22 (8.5)	35 (9.5)
HomeCARE II	07/2008–01/2012	152 (7.4)	38 (14.7)	46 (12.5)
J-HomeCARE II	06/2010–01/2012	121 (5.9)	16 (6.2)	22 (6.0)
DetectICI	12/2012–06/2016	102 (5.0)	9 (3.5)	12 (3.3)
CASTLE-AF	01/2008–03/2017	66 (3.2)	29 (11.2)	44 (11.9)
effecT	05/2008–03/2013	36 (1.8)	3 (1.2)	4 (1.1)
Total	01/2008–ongoing	2050 (100)	259 (100)	369 (100)

Study acronyms are explained in Supplementary material online, *Table S1* in the Supplementary material.

WHF, worsening heart failure.

^a^Number of patients fulfilling selection criteria (arranged in descending order).

^b^WHF hospitalizations fulfilling selection criteria.

### Follow-up duration, events, and demographics

During a median follow-up period of 643 days (IQR, 398–917 days), 259 patients had a total of 369 WHF hospitalizations fulfilling the selection criteria for this analysis. The baseline characteristics of the patients are summarized in *Table 2*. Compared with patients without WHF hospitalizations, hospitalized patients had a larger prevalence of NYHA class III (63.7% vs. 53.8%, *P* = 0.003), a slightly lower proportion of CRT-D devices (70.7% vs. 76.5%, *P* = 0.040), and a more frequent history of AF (45.4% vs. 19.3%, *P* < 0.001), stroke or transient ischaemic attack (14.4% vs. 9.5%, *P* = 0.031), renal insufficiency (33.5% vs. 18.3%, *P* < 0.001), and chronic pulmonary disease (23.6% vs. 15.7%, *P* = 0.002). In addition, hospitalized patients were more likely to be on diuretics (95.0% vs. 87.0%, *P* < 0.001), antiarrhythmic drugs (28.3% vs. 20.0%, *P* = 0.002), and digitalis (14.7% vs. 8.0%, *P* < 0.001).

**Table 2:** Baseline characteristics of patients

Parameter^a^	All patients (*n* = 2050)	Group with WHF hosp. (*n* = 259)	Group without WHF hosp. (*n* = 1791)	*P*-value^b^
Age, years	67 (59–74)	68 (62–74)	67 (59–74)	0.205
Male sex	1574 (76.8)	205 (79.2)	1369 (76.4)	0.334
Body mass index, kg/m^2^	26.9 (24.1–30.4)	26.9 (24.2–30.7)	26.9 (24.0–30.4)	0.340
New York Heart Association class				
II	922 (45.0)	94 (36.3)	828 (46.2)	0.003
III	1128 (55.0)	165 (63.7)	963 (53.8)	0.003
LVEF, %	29 (25.0–32.0)	28 (23.8–32.0)	29 (25.0–32.0)	0.164
QRS duration, ms	138 (110–162)	129 (110–159)	140 (110–162)	0.164
Primary prevention ICD indication^c^	1464 (89.8)	172 (89.6)	1292 (89.8)	0.931
CRT-D implanted	1553 (75.8)	183 (70.7)	1370 (76.5)	0.040
Ischaemic heart failure aetiology	1003 (49.0)	141 (54.4)	862 (48.2)	0.059
Hypertension	1293 (64.2)	167 (65.0)	1126 (64.1)	0.771
Systolic blood pressure	120 (110–130)	120 (109–130)	120 (110–130)	0.510
Valvular heart disease	1224 (65.1)	169 (69.3)	1055 (64.4)	0.141
Atrial fibrillation history	381 (21.4)	79 (45.4)	302 (19.3)	<0.001
History of stroke or TIA	175 (10.1)	29 (14.4)	146 (9.5)	0.031
Diabetes	751 (36.7)	107 (41.3)	644 (36.0)	0.098
Renal insufficiency	400 (20.1)	78 (33.5)	322 (18.3)	<0.001
Chronic pulmonary disease	330 (16.6)	55 (23.6)	275 (15.7)	0.002
Liver disease	52 (3.9)	9 (7.0)	43 (3.6)	0.056
Medication				
Beta-blocker	1800 (89.1)	233 (90.3)	1567 (88.9)	0.493
Diuretic	1779 (88.0)	245 (95.0)	1534 (87.0)	<0.001
ACEI or ARB	1700 (84.1)	215 (83.3)	1485 (84.2)	0.712
Antilipemic agent	1046 (62.2)	115 (65.7)	931 (61.7)	0.305
Antiplatelet	887 (44.1)	119 (47.0)	768 (43.7)	0.316
Anticoagulant	668 (33.1)	111 (43.0)	557 (31.6)	<0.001
Antiarrhythmic drug	425 (21.0)	73 (28.3)	352 (20.0)	0.002
Digitalis	179 (8.9)	38 (14.7)	141 (8.0)	<0.001
Ca^2+^ antagonist	138 (7.1)	14 (6.1)	124 (7.2)	0.552

Data are shown as median (interquartile range) or *n* (% of available data).

ACEI, angiotensin-converting-enzyme inhibitor; ARB, adenosine receptor blocker; CRT-D, cardiac resynchronization therapy defibrillator; ICD, implantable cardioverter-defibrillator; LVEF, left ventricular ejection fraction; TIA, transient ischaemic attack; WHF hosp., worsening heart failure hospitalization fulfilling selection criteria.

^a^Determined before device implantation except for ‘CRT-D implanted’.

^b^Between patients with vs. without WHFH.

^c^The remaining patients had secondary prevention indication.

### Temporal trends of HF Score and its components

**Figure 2:**
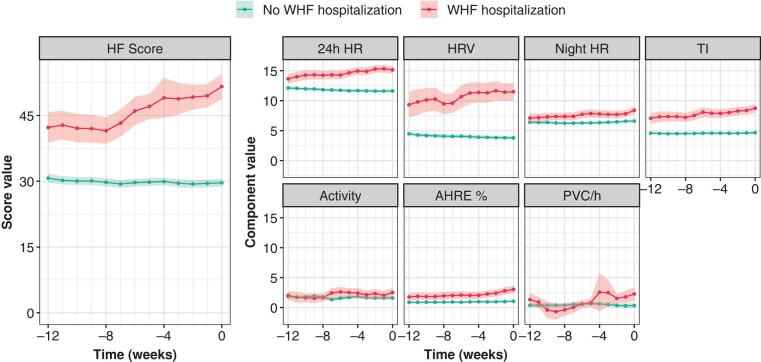
Temporal trends of weekly average values of HF Score and its components from Week −12 to Week 0 before the last Home Monitoring transmission in 1791 patients with no WHF hospitalizations and before WHF hospitalizations in 259 patients with events. The shaded areas depict the 95% confidence intervals. Components are arranged according to their contribution to the HF Score in patients with WHF hospitalizations. 24 h HR, monotone increase of 24 hour mean heart rate; Activity, trend of patient activity; AHRE %, burden of atrial high-rate episodes expressed in per cent of 24 h; HF, heart failure; HRV, monotone decrease of heart rate variability; Night HR, instability of nocturnal mean heart rate; PVC/h, trend of premature ventricular contractions per hour; TI, monotone decrease of thoracic impedance; WHF, worsening heart failure.

*Figure 2* illustrates the trends of weekly average values of the HF Score and its components during 12 weeks before WHF hospitalizations vs. 12 weeks before the last available HM message in patients free of hospitalizations. Correspondingly, *Table 3* reports the first (Week −12) and final (Week 0) values of the trendlines of *Figure 2*, along with the results of linear mixed model analysis. The HF Score was 42.3 ± 26.1 at Week −12 before WHF hospitalization vs. 30.7 ± 20.6 at Week −12 in the group with no events (*P* < 0.001, mixed-model intercept comparison). In hospitalized patients, the HF Score further increased by ∼22% between Week −12 and Week 0 to reach an average value of 51.6 ± 26.8 (*P* = 0.003, mixed-model slope comparison), whereas no increase is seen in patients without events.

**Table 3:** Comparisons of linear fits of temporal trends of HF Score and its components during 12 weeks before WHF hospitalization or last HM message in patients with no events

HF Score and its components	HF Score Week −12	HF Score Week 0	Between-group comparisons (linear mixed model analysis)	
			Week −12 before events vs. no events *P*-value^a^	Linear slope from Week −12 to Week 0 before events vs. no events *P*-value^b^
HF Score			<0.001	0.005
Before last HM message (group: no events)	30.7 ± 20.6	29.7 ± 18.9		
Before WHF hospitalizations (group: before events)	42.3 ± 26.1	51.6 ± 26.8		
HF Score components				
24 h HR			<0.001	0.82
No events	12.1 ± 5.3	11.6 ± 5.0		
Before events	13.7 ± 6.1	15.2 ± 5.8		
HRV			<0.001	<0.001
No events	4.5 ± 7.6	3.8 ± 6.1		
Before events	9.3 ± 16	11.5 ± 13.9		
Night HR			0.79	<0.001
No events	6.4 ± 4.6	6.6 ± 4.9		
Before events	7.1 ± 5.4	8.4 ± 6.1		
TI			<0.001	0.019
No events	4.6 ± 4.2	4.7 ± 4.2		
Before events	7.1 ± 6.7	8.7 ± 6.9		
Activity			0.65	0.014
No events	1.9 ± 6.5	1.6 ± 6.2		
Before events	2.0 ± 5.5	2.5 ± 6.9		
AHRE %			0.071	<0.001
No events	0.9 ± 3.5	1.0 ± 3.9		
Before events	1.8 ± 4.9	3.0 ± 6.0		
PVC/h			0.74	<0.001
No events	0.4 ± 8.8	0.3 ± 8.5		
Before events	1.3 ± 8.0	2.3 ± 10.6		

Data are mean ± standard deviation.

24 h HR, monotone increase of 24 hour mean heart rate; Activity, trend of patient activity; AHRE %, burden of atrial high-rate episodes expressed in per cent of 24 h; HF, heart failure; HM, Home Monitoring; HRV, monotone decrease of heart rate variability; Night HR, instability of night heart rate; PVC/h, trend of premature ventricular contractions per hour; TI, monotone decrease of thoracic impedance.

^a^Between-group comparison of model intercepts, representing model-predicted values at Week −12 before event (group with WHF hospitalization) or Week −12 before the last HM message (group without WHF hospitalization).

^b^Between-group comparison of model linear slopes, representing model-predicted weekly variation rate of temporal trends.

In the analysis of algorithm components (trendlines shown in the right panel of *Figure 2*; first and final average values reported in *Table 3*), either a significantly higher average value at Week −12 (24 h HR, HRV, TI; *P* < 0.001) or a significantly larger increase from Week −12 to Week 0 (all but 24 h HR, *P* < 0.02) was observed in hospitalized than in non-hospitalized patients (*Table 3*). At Week −12 before WHF hospitalizations, the 24 h HR, HRV, and TI components accounted for ∼32%, 22%, and 17% of the average score value, respectively. While Night HR, Activity, AHRE %, and PVC/h did not differ significantly at Week −12 between hospitalized and non-hospitalized patients, the slope of these components from Week −12 to Week 0 was significantly higher in hospitalized patients (*P* < 0.001 for all comparisons, except for *P* = 0.014 for Activity). The cumulative contribution of the Night HR, Activity, AHRE %, and PVC/h components to the average value of the HF Score at the time of WHF hospitalization was 31%.

**Figure 3:**
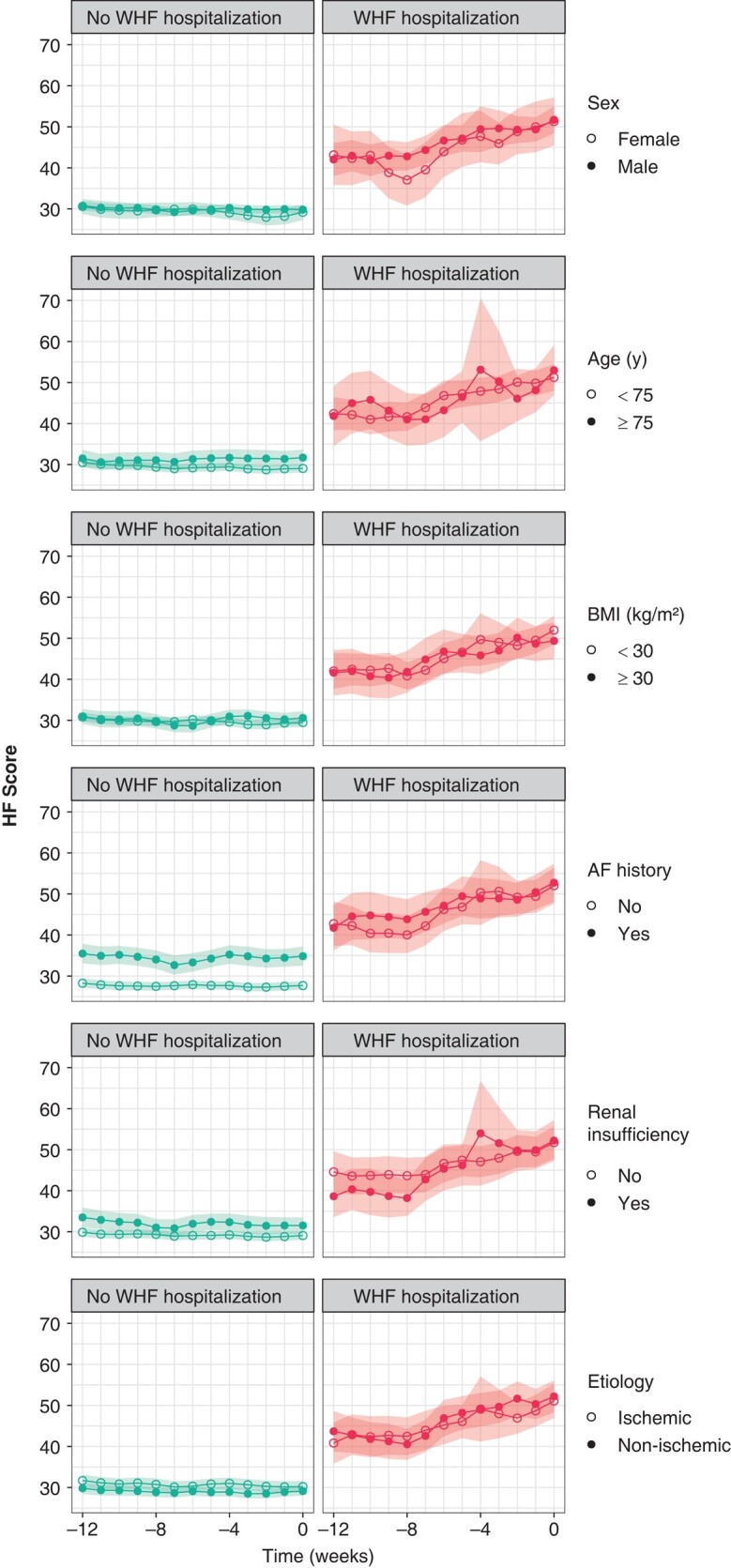
Temporal trends of average HF Score values from Week −12 to Week 0 before the last Home Monitoring transmission in patients with no WHF hospitalizations and before WHF hospitalizations in patients with events by pre-specified subgroups. Shaded areas depict the 95% confidence intervals. AF, atrial fibrillation; BMI, body mass index; HF, heart failure; WHF, worsening heart failure.

The HF Score had similar trends in all subgroups based on patient characteristics (*Figure 3*). In particular, the linear models of HF Score trends before WHF hospitalizations did not differ significantly between the subgroups, except for a slightly higher slope in the ischaemic subgroup (*P* = 0.04; *Table 4*).

**Table 4:** Comparisons of linear fits of temporal trends of HF Score 12 weeks before WHF hospitalization by pre-specified patient subgroups

Patient subgroups	HF Score Week −12	HF Score Week 0	Comparison between patient subgroups (linear mixed model analysis)	
			Week −12 *P*-value	Linear slope from Week −12 to Week 0 *P*-value
Sex			0.54	0.30
Male (*n* = 205)	42.0 ± 26.4	51.7 ± 27.2		
Female (*n* = 54)	43.2 ± 25.3	51.3 ± 25.4		
Age			0.23	0.12
<75 years (*n* = 196)	42.4 ± 26.3	51.2 ± 27.1		
≥75 years (*n* = 63)	41.8 ± 25.5	53.0 ± 25.9		
BMI			0.56	0.08
<30 kg/m^2^ (*n* = 175)	42.0 ± 26.6	52.1 ± 26.7		
≥30 kg/m^2^ (*n* = 79)	41.6 ± 22.8	49.4 ± 24.0		
AF history			0.36	0.59
No (*n* = 136)	42.8 ± 29.2	52.0 ± 29.1		
Yes (*n* = 87)	41.8 ± 23.1	52.7 ± 24.9		
Renal insufficiency			0.35	0.48
No (*n* = 155)	44.6 ± 29.0	51.7 ± 28.2		
Yes (*n* = 78)	38.6 ± 20	52.2 ± 26.2		
Aetiology			0.49	0.040
Non-ischaemic (*n* = 118)	43.7 ± 26.2	52.2 ± 25.4		
Ischaemic (*n* = 141)	40.8 ± 26.0	51.1 ± 28.1		

Data are mean ± standard deviation.

AF, atrial fibrillation; BMI, body mass index; HF, heart failure; WHF, worsening HF.

The HF Score trends did not differ between patients with vs. without AF history (*P* = 0.36 for score values and *P* = 0.59 for trend slopes). This suggests a similar algorithm prediction performance in both subgroups despite a higher HF Score in the AF subgroup also in non-hospitalized patients (average 35.5 vs. 28.3, *P* < 0.001; *Figure 3*). The higher HF Score values in the subgroup with AF history were mainly the consequence of a greater contribution of the AHRE % component to the HF score, as illustrated in Supplementary material online, *Figure S1* of the Supplementary material.

## Discussion

In a pooled dataset of 2050 patients from nine clinical trials, we compared temporal trends of the HeartInsight HF Score before WHF hospitalizations with trends in patients without events. Irrespective of algorithm programming and alert timing, the HF Score showed a significantly increasing trend from Week −12 to Week 0 before WHF hospitalization, in contrast to constant values in patients without events. Since the score is updated daily by a numerical processing of several physiologic parameters, these findings support the hypothesis that the score temporal trend incorporates information linked to the patient’s HF status and that an increasing trend may reflect worsening HF conditions regardless of the alert status. The culmination of these trends over time is what triggers the alert when the HF Score exceeds the programmed threshold. On average, the default threshold (45) is reached 6 weeks before hospital admission (*Figure 2*), which is consistent with the results of the Selene HF trial. Thereafter, the patient status should be assessed, as proposed by Zanotto and Capucci.^10^

It has been shown that WHF manifests itself with a large variety of severity degrees and symptoms.^8^ Hospitalization is one of possible outcomes of decompensation processes in most severe or unsupervised cases, but timing and sometimes even occurrence of hospital admissions depend on several factors, including availability of in-person or remote medical counselling and psychological factors.^11^ In our analysis, the HF Score increased significantly before a WHF hospitalization. Overall, these results suggest that the HF Score should not be regarded as a mere alert system. The temporal trend may be considered as a continuous quantitative estimation of HF conditions and their evolution, regardless of patient symptoms. This is important, to give clinical meaning to the HF Score during its use in medical practice. In a recent scientific statement on remote monitoring for HF management at home, Stevenson *et al*.^12^ suggest that many of ‘false-positive alerts’ regarding WHF hospitalization may actually be true positives for the patient, bringing attention to conditions that warrant diagnosis and therapy. Findings of changes over time in the cardiovascular status of the patient as reflected by the HF Score trendline highlight the importance of assessing the patient soon after an alert has been triggered.

As shown by the SELENE HF trial, dynamic, timing, and amplitude of changes differ among the variables processed by the algorithm. According to the analysis of component weights reported in *Table 3*, 24 h HR, HRV, and Night HR collectively accounted for an average of 71% of the HF Score 12 weeks prior to WHF hospitalization. This confirms the central role of heart rate components in HF monitoring.^12,13^ However, the behaviour of these three components differed in the analysed period: whereas the 24 h HR and HRV components were already higher at Week −12 in hospitalized vs. non-hospitalized patients, the HRV component increased and 24 h HR did not increase significantly until the hospitalization. This may be explained by the design of the algorithm in which the value of the HF Score is increased when a continuous increase in daily average heart rate is detected. As heart rate cannot increase without physiological limitations, the corresponding algorithm component cannot increase any further. Our analysis revealed that the plateau is reached on average 12 weeks before WHF hospitalization (*P* = 0.82 for slope comparison between patients with and without events). Whether this is caused by earlier increase or initial higher values reflecting worse baseline conditions, needs to be assessed by specifically designed analyses (manuscript in preparation). In all cases, the 24 h HR represents a long-term monitoring and therapeutic target.^14^ Conversely, the Night HR component was similar at Week −12 in patients with and without events, but it increased significantly during 12 weeks before the event. This is consistent with a correlation of the night heart rate instability with acute decompensation over a shorter term. Since night heart rate has been shown to be stronger predictor of total mortality and ventricular arrhythmias than the 24 h HR,^15^ an increase of this algorithm component should prompt faster evaluation.

The Activity and PVC/h components showed similar values in patients with vs. without events at Week −12. Both components increased significantly until WHF hospitalization. The TI component had a significantly higher value at Week −12 in patients with events and continued to increase until hospitalization. Since an increasing TI component results from decreasing periods in the underlying TI measurement, an upward trend in the HF Score with a simultaneous downward trend in TI should attract attention.

A special consideration merits the AHRE % component. Its average contribution to the HF Score was 6% overall, likely explained by a rapid deteriorating effect of AF on HF status, which makes AF less helpful for prediction purpose.^16^ However, our subgroup analysis revealed that in patients with AF history, the HF Score value was on average higher than in patients with no AF history, even in the absence of HF events. This suggests that the AHRE % component is one of the main drivers of the HF Score increase before a WHF hospitalization in patients with AF history (see Supplementary material online, *Figure S1* in the Supplementary material). In these patients, AF may be the predominant mechanism of decompensation, and control of their cardiac rhythm may be relevant to controlling risk of WHF hospitalization.^17^

It is important to note that the HF Score in the 12 weeks prior to WHF hospitalization showed similar increasing trends in all the pre-specified subgroups, including similar initial and final values. This is encouraging because similar performance may be expected from the HF Score regardless of the main patient characteristics that we considered in our analysis. It should be emphasized that these results have been obtained excluding the Seattle HF Model score, whose inclusion in the model aimed to better match the performance of the HF Score to the patient profile.

### Other multiparameter algorithms for worsening heart failure prediction

The seven longitudinal HM parameters used by the HeartInsight algorithm to generate the HF Score were selected because they are known to be related to patient HF status at varying degrees of correlation.^18^ Other multiparameter algorithms for WHF prediction include some different parameters, such as mathematical inference of the first and third heart sound, respiration rate, and ratio of respiration rate to tidal volume, on top of thoracic impedance, patient activity, and heart rate (HeartLogic™ HF diagnostic by Boston Scientific, St. Paul, MN, USA).^19–23^ Another example is the use of OptiVol^®^ fluid index (derived from thoracic impedance) on top of HRV, night heart rate, patient activity, and a combined heart rhythm parameter comprising AF burden, ventricular rate during AF, treated ventricular tachyarrhythmia, and percentage of CRT pacing (Triage HF Risk Status by Medtronic Inc., Minneapolis and Tempe, USA).^23–27^ Implemented on different remote monitoring platforms, these systems have been introduced for intensified monitoring and management of HF patients, with varying degrees of success.^19–29^

In addition to the multiparameter algorithms used in ICD and CRT-D patients, a dedicated implantable diagnostic device capable of measuring only pulmonary artery pressure has proved useful in predicting and preventing WHF hospitalizations.^3,30^ As this sensor cannot be integrated into implantable therapeutic cardiac devices, they continue to rely on multiparameter algorithms.

The results of our analysis add information on trends and weight of individual components of a multiparameter WHF predictor, which can facilitate interpretation and use in medical practice. However, to assess the clinical benefit of such tools in the management of HF patients with reduced ejection fraction, randomized outcome trials are necessary.

### Study limitations

This is a retrospective analysis of nine clinical trials spanning a wide period (from the CASTLE-AF trial initiated in 2008 to the ongoing BIO|Stream.HF) and encompassing important developments in HF therapy and management over the years, including the introduction of sodium-glucose co-transporter-2 inhibitors and mineralocorticoid receptor antagonists (not specifically reported here). This certainly led to some heterogeneity in the cohort included in the analysis and in the pool of endpoint events. Inevitable differences in data collection methods between studies also resulted in the exclusion of many patients, as explained above, or missing data, e.g. those needed to calculate the Seattle HF Model score. This prevented a full evaluation of algorithm performance. Nevertheless, the heterogeneity of the pooled datasets supports the generalizability of results.

Based on the manufacturer’s instructions for use of the HeartInsight algorithm, we excluded patients with long-standing or permanent AF, LVEF > 35%, or NYHA class other than II/III, which are usually encountered in clinical practice. AF also prevents the algorithm from processing the HRV component, which according to our analysis is one of the main contributors.

Finally, we analysed HF Score and component trends before a relatively large number of WHF hospitalizations. However, since we could not continuously track patients’ HF conditions during follow-up, we were unable to assess whether similar trends would also occur when the condition worsened without hospitalization. Therefore, our differential analysis of the HF Score and contributions concerns only their sensitivity and not their specificity.

## Conclusions

In a pooled dataset of more than 2000 patients, the HeartInsight HF Score showed a significantly increasing trend during 12 weeks before WHF hospitalization. These findings support the clinical relevance of the HF Score as a quantitative estimate of the patient’s HF condition prior to crossing the nominal threshold. Examination of specific variable values and their trends, in addition to the temporal trends of the combined HF Score, may provide valuable information to monitor patient’s condition and facilitate interpretation, medical decision-making, and action timing.

## Supplementary Material

euae032_Supplementary_Data

## Data Availability

The data underlying this article were provided by Biotronik SE & Co. KG. Data will be shared on request to the corresponding author with permission of Biotronik SE & Co. KG.
