# Supplementary material for: Predicting worsening heart failure hospitalizations in patients with implantable cardioverter defibrillators: is it all about alerts? A pooled analysis of nine trials
Source: Europace. 2024 Jan 31;26(2):euae032. doi: 10.1093/europace/euae032 (PMC10858640; doi:10.1093/europace/euae032)
Supplement: euae032_Supplementary_Data [file euae032_supplementary_data.docx]

**Supplementary appendix**

**Contents**

Supplementary tables and figures

Table S1 …..........................................…………………………………………………….. page 2

Figure S1 .............................................…………………………………………………… page 5

References …………………………………………………………………………………………………. page 6

**Table S1** Details of clinical trials included in data analysis (alphabetical order)

| **Clinical trial** | **Trial details** |
| --- | --- |
| **Completed trials** |  |
| CASTLE-AF^1^ | ClinicalTrials.gov number: NCT00643188 |
| Study name | Catheter ablation versus standard conventional therapy in patients with LV dysfunction and AF |
| Design | Randomized 1:1 (catheter ablation vs. standard conventional therapy) |
| Centers | 33 sites (Europe, Australia, United States) |
| Number of patients | 363 randomized |
| Enrolment years | 2008 – 2016 |
| Mean follow-up | 37.6 months |
| Primary endpoint | A composite of death from any cause or WHF leading to an unplanned overnight hospitalization |
| Main inclusion | Paroxysmal or persistent AF; an absence of response to, unacceptable side effects from, or unwillingness to take antiarrhythmic drugs; NYHA class II, III, or IV HF with a LVEF ≤35%; ICD or CRT-D with HM |
| Main exclusion | Candidacy for heart transplantation or planned cardiovascular intervention |
| DetectICI^a^ | Registered at Japanese Primary Registries Network, ID number: 000009660 |
| Study name | A Prospective, non-randomized, multicenter study to demonstrate the clinical validity of the intracardiac impedance (ICI) measurements by comparing the ICI values with clinical markers which are relevant for an efficient therapy management of HF patients |
| Design | Observational |
| Centers | 38 sites (Japan) |
| Number of patients | 198 |
| Enrolment years | 2012 – 2015 |
| Mean follow-up | 10.8 months |
| Primary objective | To investigate the relationship between end-systolic ICI and echocardiographic LV end-systolic volume |
| Main inclusion | Standard indication for CRT-D therapy, a new bipolar LV lead implanted with a tip-ring distance of ≥15 mm, a true bipolar right ventricular shock lead implanted, acceptance of HM |
| Main exclusion | Permanent AF and a contraindication for programming a mode other than DDD, aortic valve stenosis, severe aortic insufficiency, aortic valve prosthesis, cardiac surgery planned or done within the previous 3 months, or chronic renal dialysis |
| EchoCRT^2^ | ClinicalTrials.gov number: NCT00683696 |
| Study name | [Echocardiography guided cardiac resynchronization therapy](https://clinicaltrials.gov/study/NCT00683696?term=EchoCRT%20biotronik&rank=1) |
| Design | Randomized 1:1 (CRT capability turned on vs. CRT capability turned off) |
| Centers | 115 sites (United States, Canada, Israel, Australia, Europe) |
| Number of patients | 809 randomized |
| Enrolment years | 2008 – 2013 |
| Mean follow-up | 19.4 months |
| Primary endpoint | Combination of death from any cause or first hospitalization for WHF (efficacy outcome). Freedom from complications related to the CRT-D system at 6 months (safety outcome) |
| Main inclusion | NYHA class III or IV HF, LVEF ≤35%, standard ICD indication, stable medical therapy recommended by current guidelines, QRS duration <130 msec, LVEDD ≥55 mm, echocardiographic evidence of LV dyssynchrony |
| Main exclusion | Acute decompensated HF, intravenous inotropic therapy, AF within the previous month, or bradycardia requiring pacing |
| ECOST-CRT^a^ | ClinicalTrials.gov number: NCT03012490 |
| Study name | Efficacy, safety, and cost of comprehensive remote monitoring versus standard remote monitoring in patients with CRT |
| Design | Randomized 1:1 (remote monitoring vs. ambulatory follow-ups) |
| Centers | 45 sites (France) |
| Number of patients | 652 randomized |
| Enrolment years | 2017 – 2020 |
| Mean follow-up | 18 months |
| Primary endpoint | Composite of death from any cause or worsening HF hospitalization, whichever comes first |
| Main inclusion | Implanted with CRT-P or CRT-D device with HM activated |
| Main exclusion | Life expectancy < 1 year, planned for ventricular assistance |
| effecT^a^ | ClinicalTrials.gov number: NCT00811382 |
| Study name | Clinical effect of HF management via HM with a focus on AF |
| Design | Randomized 1:1 (full access vs. limited access to HM service center) |
| Centers | 21 sites (Europe, 7 countries) |
| Number of patients | 163 randomized |
| Enrolment years | 2008 – 2012 |
| Mean follow-up | 11.4 months |
| Primary endpoint | Clinical composite outcome based on the days lost due to cardiovascular mortality, cardiovascular hospitalization and inappropriate ICD therapy during an observational period of 12 months |
| Main inclusion | Indication for CRT-D, paroxysmal or persistent AF, optimized HF-related medication |
| Main exclusion | Permanent AF, contraindication for anticoagulation, stroke within the last 6 weeks, or acute coronary syndrome within the last 2 months |
| HomeCARE II^3^ | ClinicalTrials.gov number: NCT00711360 |
| Study name | Monitoring of fluid status in HF patients by intrathoracic impedance measurement |
| Design | Interventional |
| Centers | 33 sites (Europe and Israel) |
| Number of patients | 303 |
| Enrolment years | 2008 – 2009 |
| Mean follow-up | 22.4 months |
| Primary endpoint | Clinically apparent HF events used to develop an intrathoracic impedance-based algorithm for the prediction of imminent cardiac decompensation |
| Main inclusion | Eligible for 1-, 2- or 3-chamber ICD implantation or already implanted with a Lumax 540 device or successor; NYHA class II, III, or IV; LVEF ≤35%; acceptance of HM |
| Main exclusion | Acute coronary syndrome within the last 3 months, actively listed for heart transplantation, post heart transplantation, cardiac surgery planned or done within the previous 3 months, or chronic renal dialysis |
| J-HomeCARE II^3^ | ClinicalTrials.gov number: NCT01221649 |
| Study name | Monitoring of fluid status in HF patients by intrathoracic impedance measurement in Japan |
| Design | Interventional |
| Centers | 25 sites (Japan) |
| Number of patients | 198 |
| Enrolment years | 2010 – 2012 |
| Mean follow-up | 19.4 months |
| Primary endpoint | Clinically apparent HF events used to develop an intrathoracic impedance-based algorithm for the prediction of imminent cardiac decompensation |
| Main inclusion | Eligible for 1-, 2- or 3-chamber ICD implantation or already implanted with a Lumax 540 device or successor; NYHA class II, III, or IV; LVEF ≤35%; acceptance of HM |
| Main exclusion | Acute coronary syndrome within the last 3 months, actively listed for heart transplantation, post heart transplantation, cardiac surgery planned or done within the previous 3 months, or chronic renal dialysis |
| SELENE HF^4^ | ClinicalTrials.gov number: NCT01836510 |
| Study name | Selection of potential predictors of WHF |
| Design | After prospective collection of data, randomization 1:1 for data processing (predictive algorithm derivation cohort vs. algorithm validation cohort) |
| Centers | 34 sites (Italy, Spain) |
| Number of patients | 918 randomized |
| Enrolment years | 2012 – 2017 |
| Median follow-up | 22.5 months |
| Primary endpoint | The ﬁrst post-implant adjudicated HF hospitalization |
| Main inclusion | Patients with an CRT-D or ICD capable of atrial sensing, HM feature, LVEF ≤35%, NYHA class II or III |
| Main exclusion | Permanent AF, acute HF, previous stroke, planned cardiac surgery |
| **Ongoing trials** |  |
| BIO\|Stream.HF^5^ | ClinicalTrials.gov number: NCT03366545 |
| Study name | Observation of clinical routine care for HF patients implanted with BIOTRONIK CRT devices |
| Design | Observational |
| Centers | 118 sites recruiting currently (Australia, Japan, Taiwan, Singapore, Israel, South Africa, Europe) |
| Number of patients | Estimated enrolment: 3000 |
| Enrolment years | 2018 – ongoing |
| Target follow-up | 3 years |
| Primary endpoint | No primary endpoints defined. |
| Main inclusion | Planned de novo implantation of or upgrade to a CRT system, acceptance of HM |
| Main exclusion | Contraindication for CRT device implantation |

^a^No publication available.

Abbreviations: AF, atrial fibrillation; CRT, cardiac resynchronization therapy; CRT-D, CRT defibrillator; HF, heart failure; HM, Biotronik Home Monitoring feature; ICD, implantable cardioverter-defibrillator; ICI, intracardiac impedance; LV, left ventricular; LVEDD, LV end-diastolic diameter; LVEF, LV ejection fraction; NYHA, New York Heart Association; WHF, worsening HF.


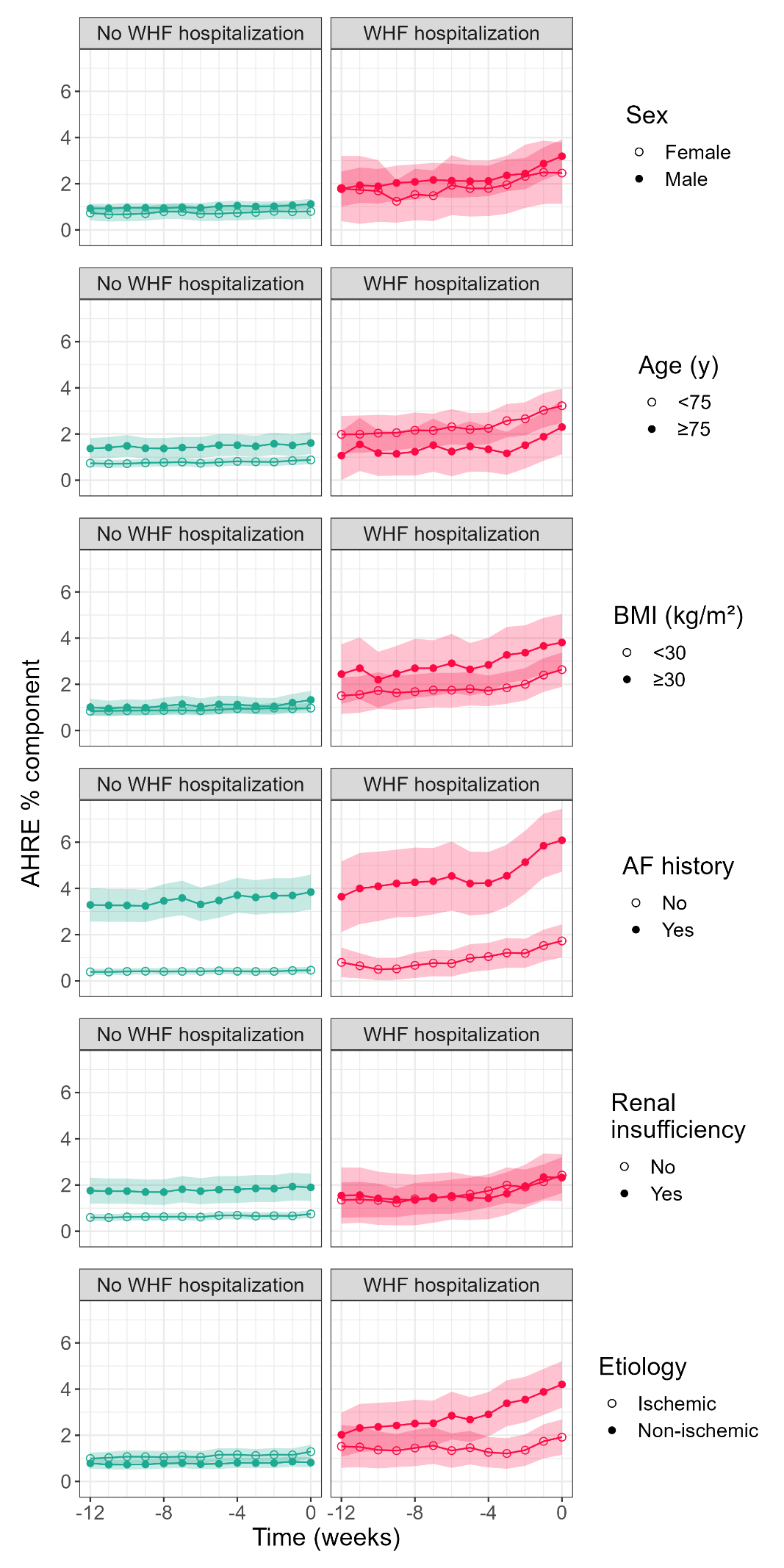


**Figure S1.** Temporal trends of the AHRE % component of the HF score before WHF hospitalizations (patients with events) and before the last HM message (patients without events) by prespecified subgroups. Shaded areas depict the 95% confidence intervals. The AHRE % component was significantly higher in the subgroup with vs. without AF history (p<0.001 for comparison of values at week -12; p=0.007 for comparison of the increasing trend until the event). Abbreviations: AF, atrial fibrillation; BMI, body mass index; HF, heart failure; WHF, worsening heart failure.

**References**

1. Marrouche NF, Brachmann J, Andresen D, Siebels J, Boersma L, Jordaens L, et al. Catheter ablation for atrial fibrillation with heart failure. *N Engl J Med* 2018; 378:417-27.

2. Ruschitzka F, Abraham WT, Singh JP, Bax JJ, Borer JS, Brugada J, et al. Cardiac-resynchronization therapy in heart failure with a narrow QRS complex. *N Engl J Med* 2013; 369:1395-405.

3. Maier SKG, Paule S, Jung W, Koller M, Ventura R, Quesada A, et al. Evaluation of thoracic impedance trends for implant-based remote monitoring in heart failure patients - results from the (J-)HomeCARE-II study. *J Electrocardiol* 2019; 53:100-8.

4. D'Onofrio A, Solimene F, Calo L, Calvi V, Viscusi M, Melissano D, et al. Combining home monitoring temporal trends from implanted defibrillators and baseline patient risk profile to predict heart failure hospitalizations: results from the SELENE HF study. *Europace* 2022; 24:234-44.

5. Schmitt J, Wenzel B, Brüsehaber B, Anguera I, de Sousa J, Nölker G, et al. Impact of lockdown during COVID-19 pandemic on physical activity and arrhythmia burden in heart failure patients. *Pacing Clin Electrophysiol* 2022; 45:471-80.
